# Interobserver Reliability among Radiologists and Orthopaedists in Evaluation of Chondral Lesions of the Knee by MRI

**DOI:** 10.4061/2011/743742

**Published:** 2011-07-07

**Authors:** Fábio Cavalli, Anela Izadi, Ana Paula R. B. Ferreira, Larissa Braga, Andresa Braga-Baiak, Marco Antonio Schueda, Mihir Gandhi, Ricardo Pietrobon

**Affiliations:** ^1^Clinical Research Division, Orthopedics and Traumatology Institute (IOT), Brazil Rua Blumenau, 1316 América Joinville, 89204-251 Joinville, SC, Brazil; ^2^Research on Research Group, Duke University, Durham, P.O. Box 3094, NC 27710, USA; ^3^Univille University, Joinville, Brazil Univille Universidade Bairro Bom Retiro Campus Universitário, 89219-950 Joinville, SC, Brazil; ^4^Singapore Clinical Research Institute, Singapore 31 Biopolis Way, Nanos #02-01, Singapore 138669; ^5^Duke-NUS Graduate Medical School, Singapore 8-College Road, SGH Outram Campus, Singapore 169857; ^6^Duke University Health System, Durham, P.O. Box 3094, NC 27710, USA

## Abstract

*Objective*. The aim of this study was to evaluate interobserver reliability in the presence of chondral injuries of the knee among radiologists, orthopaedic surgeons, radiologists, and orthopaedic surgeons. *Methods*. This was a prospective, web-based multi-institutional survey, consisting of 6 magnetic resonance exams of knee chondral injuries and a questionnaire to be completed by the participants. Two radiologists and two orthopaedic surgeons were enrolled, with more than 5 years of clinical experience. Kappa statistics test was used to calculate interobserver reliability between participants. *Results*. Kappa ranged from −0.13 through 0.29 between orthopaedists; from 0.06 through 0.78 between radiologists; from −0.10 through 0.24 between orthopaedists and radiologists. Cases 3 and 6 had skewed results among radiologists: with Kappa scores of 0.78 and 0.53, respectively. *Conclusions*. Our study reveals that the interobserver agreement between radiologists is higher than among orthopaedists in the evaluation of chondral knee lesions by MRI.

## 1. Introduction

Advancements in diagnostic imaging and the development of minimally invasive surgical techniques have enhanced the diagnosis of cartilaginous lesions, [1, 2]. Patients with chondral lesions of the knee often present with functional limitations and reduced physical activity, as a result of pain and haemarthrosis [3]. In the United States, mainly because of increased longevity and active lifestyles, more than 39 million physician visits and 500,000 hospitalizations occur each year for the treatment of degenerative articular cartilage diseases [4].

Magnetic resonance imaging (MRI) is the gold standard for the evaluation of cartilaginous and meniscal lesions, ligament integrity, and bone marrow oedema [5]. MRI is a noninvasive diagnostic tool with multiplanar capabilities, exceptional soft tissue resolution, and moderate sensitivity (42–77%), and high specificity (80–92%) to detect chondral lesions [6, 7]. Thus, MRI has become an integral resource for preoperative planning and post-operative management of numerous orthopaedic conditions, such as chondral lesions. However, the lack of standardised interpretations of chondral lesions by MRI may result in disagreements between physicians from different specialties, leading to conflicting diagnoses and treatment strategies. Musculoskeletal MR exams are interpreted by both radiologists and orthopaedic surgeons in several institutions worldwide [8, 9]. Our hypothesis is that sole radiologists would have a higher interreader agreement than orthopaedic surgeons on the evaluation of chondral lesions by MRI, when orthopaedic surgeons do not have access to patient histories and physical examination results.

## 2. Methods

### 2.1. Study Design

A prospective, web-based, multi-institutional survey was conducted after receiving approval from Institutional Review Board committee. Potential study participants were contacted via e-mail, after their names and e-mail addresses were obtained from radiological and orthopaedic societies. Consent for the study was waived, and no compensation was provided to the study participants. All identifiable health information was electronically stored on the principal investigator's password-encrypted computer, and was available only to personnel directly involved with the study. After the potential study participants were contacted, all identifiable health information data was erased. As part of an internet survey designed to assess interobserver reliability, 2 radiologists and 2 orthopaedic surgeons, board certified with more than 5 years of clinical experience, individually interpreted 6 MRIs depicting chondral lesions of the knee. The internet survey was created using Dados-Survey, which is a tool used to validate scales, (Atashili et al. [10]). After reviewing each MRI and submitting a report, the physicians were directed to an on-line questionnaire. Study participants were not given access to the review ratings at any time.

### 2.2. Imaging Techniques

**Figure 1:**
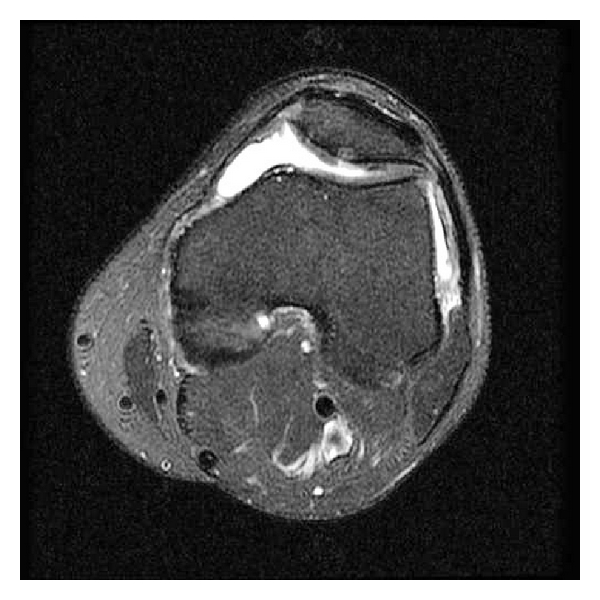
Axial T2 weighted image with fat suppression reveals a deep fissure in the patellar apex with subchondral bone reactive changes. Condropathy can also be identified in the medial patellar facet, without reactive bone changes.

**Figure 2:**
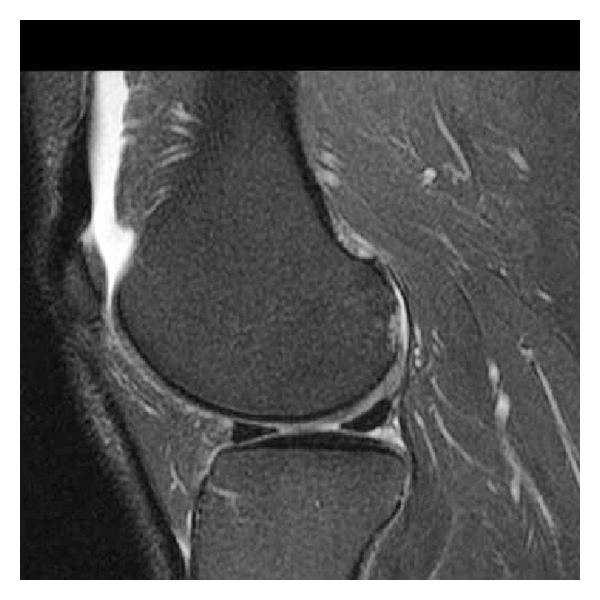
Sagittal T2 weighted image with fat suppression demonstrates the presence of a chondral defect in the posterior aspect of the lateral femoral condyle, with subchondral bone reactive changes.

**Figure 3:**
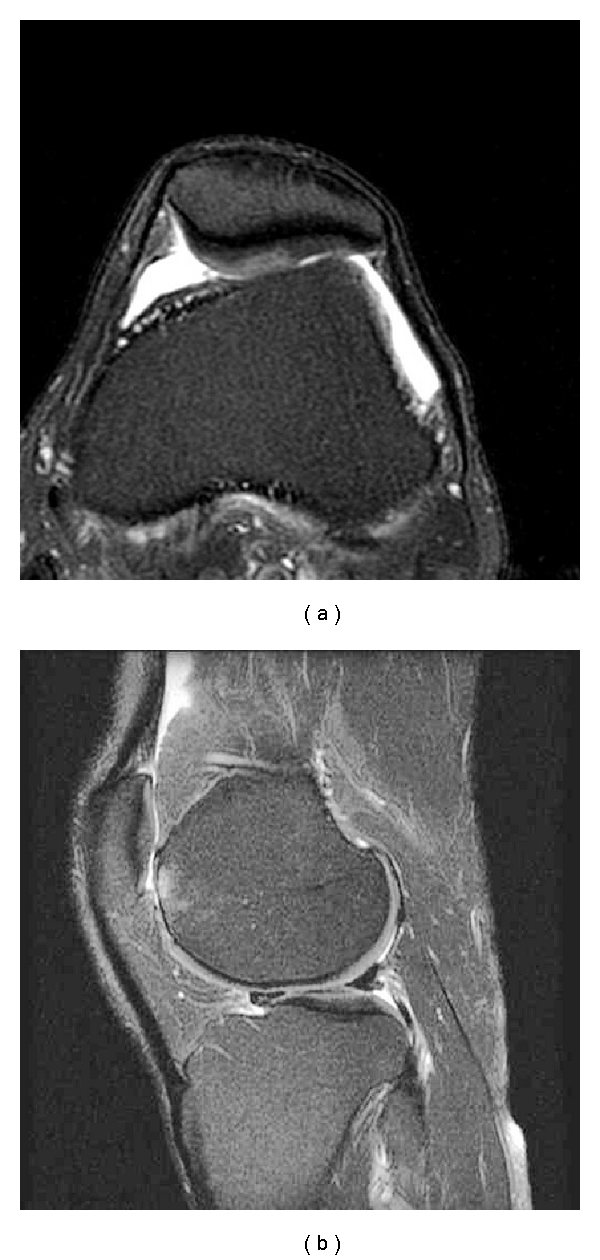
(a) Axial T2 weighted image with fat suppression depicts an area of chondral oedema characterised by an ill-defined high signal intensity focus in the lateral patellar facet. (b) In this same case, a sagittal T2 weighted image with fat suppression demonstrates the presence of a chondral defect in the lateral trochlea with reactive subchondral bone changes.

Six MR cases, previously diagnosed as chondral lesions by arthroscopy of the knee, were retrospectively selected by an independent musculoskeletal radiologist at a renowned US institution. MR exams were performed using a 1.5-T magnet (Signa; GE Medical Systems, Milwaukee, Wis, USA) using a commercially available knee coil from the same manufacture. MRIs of the knee were acquired in axial, coronal, and sagittal planes, with T2-weighted, and fat-suppressed sequences. Each chondral lesion was anatomically categorised as medial and lateral femoral condyle; medial and lateral tibial condyle; medial and lateral patellar facet; medial and lateral trochlea; patellar apex lesion. The grading system for the chondral lesions was as follows: grade 0 = normal; grade 1 = with chondral edema; grade 2 = with superficial fissures (<50%); grade 3 = with deep fissures (>50%, without bone reactive changes); grade 4 = with cartilage defects and bone reactive changes; grade 5 = with cartilage and bone defects; grade 6 = with chondral delamination (Figures 1, 2, and 3) [11].

### 2.3. Statistical Analysis

The interobserver agreement of orthopaedists, radiologists, and orthopaedists and radiologists, was evaluated using Cohen's Kappa statistics [12]. Each Kappa statistic was tested for Kappa = 0, and *P* values <0.05 were considered to be statistically significant. If a calculated Kappa statistic was found to be 0 the corresponding *P*-value was marked as “Not Applicable”. In addition, if a reader response was missing, calculation of test statistics for weighted Kappa coefficients was not possible: the corresponding *P* value was then reported as “Not Calculable”. Weighted Kappa statistics were defined as follows: Kappa < 0 was considered to indicate “no agreement”; Kappa = 0.0 to 0.20 as “slight agreement”; Kappa = 0.21 to 0.40 as “fair agreement”; Kappa = 0.41 to 0.60 as “moderate agreement”; Kappa = 0.61 to 0.80 as “substantial agreement”; Kappa = 0.81 to 1.00 as “almost perfect agreement” [13]. Statistical analyses were performed using Stata/MP 10.1 (Stata Corp, College Station, Tex).

## 3. Results

Results are shown in Table 1. Kappa values ranged from −0.13 (no agreement) through 0.29 (fair agreement) between orthopaedists; from 0.06 (slight agreement) through 0.78 (substantial agreement) between radiologists; from −0.10 (no agreement) through 0.24 (fair agreement) between orthopaedists and radiologists. Case  3 and Case  6 had skewed results, with high Kappa values between radiologists: 0.78 (substantial agreement) and 0.53 (moderate agreement), respectively.

## 4. Discussion

The most widely used classification system for chondral damage is the Outerbridge Classification system, which has been described in over 31,000 articles worldwide [14]. To the best of our knowledge, the present study is the first to evaluate interobserver agreement images of chondral lesions of the knee by MRI among orthopaedists, radiologists, and between orthopaedists and radiologists. Overall, our results suggest that there is “fair agreement” between orthopaedists; “fair agreement” between radiologists and orthopaedists; “substantial agreement” between radiologists. 

Cases  3 and  6 demonstrated skewed Kappa results between radiologists and orthopaedists. We believe the unusual abnormality location and the mild degree of some lesions on cases  3 and 6 contributed to the skewed kappa between readers from different specialties. Cases  1, 2, 4, and 5 represented a common OA pattern. Case  3 demonstrated an atypical OA with involvement of medial patella and lateral femoral condyle. Three foci of chondral edema were identified; however, two of them were mild and the lesions were overlooked by one of the orthopaedists. The patella abnormality was very subtle; meanwhile the lesion on femoral condyle was moderate/severe however the latter on atypical location. Case  6 demonstrated a tricompartmental OA but without chondral delamination. Radiologists were able to acknowledge the degree of chondral abnormality; which did not happen among orthopaedists. 

The number of studies evaluating interobserver reliability among orthopaedic surgeons is limited. von Engelhardt et al. [15] reported “moderate” interobserver agreement between orthopaedic surgeons (Kappa = 0.51–0.75) during evaluation of degenerative cartilage changes by MRI in patients diagnosed with knee osteoarthritis. As expected, radiologists solely demonstrated a higher interreader agreement when compared to orthopaedic surgeons, and radiologists and orthopaedic surgeons combined. In the clinical setting, the orthopaedic surgeons solely may have a higher interreader agreement than radiologists solely, however in the majority of the time, the formers are not blinded to clinical history and physical exam; not mentioning to arthroscopy results. Although our study is somewhat limited because of the number of MR cases and subjects included, and the non-standardised monitor image quality from the reading stations utilised, we feel that the interobserver reliability data presented in this study accurately represents MRI interpretation differences among radiologists and orthopaedists. These differences may be attributed to varying levels of experience in MRI interpretation, and specifically, interpretation of MR images of chondral lesions. Therefore, to ensure uniform evaluation methods and data reporting, seeking for the best of patient care, it is essential to follow prevalidate scales mainly if there is a report divergence between radiologists and orthopaedic surgeons.

## Figures and Tables

**Table 1:** Kappa statistics among orthopaedists, radiologists, and between orthopaedists and radiologists.

	Case = 1 *K* (*P* value)	Case = 2 *K* (*P* value)	Case = 3 *K* (*P* value)	Case = 4 *K* (*P* value)	Case = 5 *K* (*P* value)	Case = 6 *K* (*P* value)	All cases *K* (*P* value)
Orthopaedists	−0.13 (0.646)	0.31 (0.171)	0.02 (0.330)	0.29 (0.009)	0.15 (0.193)	0.11 (0.147)	0.22 (0.008)

Radiologists	0 (NA)	0.20 (0.050)	0.78 (<0.001)	0.27 (0.055)	0.06 (0.365)	0.53 (0.002)	0.37 (<0.001)

Orthopaedists and radiologists	−0.10 (0.809)*	0.24 (0.009)	0.06 (NC)	0.09 (0.146)	−0.06 (0.787)	0.15 (0.021)	0.17 (NC)**

NA: Not applicable.

NC: Not calculable due to missing response by a rater.
